# Managing the deluge of newly discovered plant viruses and viroids: an optimized scientific and regulatory framework for their characterization and risk analysis

**DOI:** 10.3389/fmicb.2023.1181562

**Published:** 2023-05-30

**Authors:** Nuria Fontdevila Pareta, Maryam Khalili, Ayoub Maachi, Mark Paul S. Rivarez, Johan Rollin, Ferran Salavert, Coline Temple, Miguel A. Aranda, Neil Boonham, Marleen Botermans, Thierry Candresse, Adrian Fox, Yolanda Hernando, Denis Kutnjak, Armelle Marais, Françoise Petter, Maja Ravnikar, Ilhem Selmi, Rachid Tahzima, Charlotte Trontin, Thierry Wetzel, Sebastien Massart

**Affiliations:** ^1^Plant Pathology Laboratory, Gembloux Agro-Bio Tech, University of Liège, Gembloux, Belgium; ^2^Univ. Bordeaux, INRAE, UMR BFP, Villenave d'Ornon, France; ^3^EGFV, Univ. Bordeaux, INRAE, ISVV, Villenave d’Ornon, France; ^4^Abiopep S.L., Murcia, Spain; ^5^Department of Biotechnology and Systems Biology, National Institute of Biology, Ljubljana, Slovenia; ^6^College of Agriculture and Agri-Industries, Caraga State University, Butuan, Philippines; ^7^DNAVision (Belgium), Charleroi, Belgium; ^8^School of Natural and Environmental Sciences, Faculty of Science, Agriculture and Engineering, Newcastle University, Newcastle upon Tyne, United Kingdom; ^9^Department of Stress Biology and Plant Pathology, Center for Edaphology and Applied Biology of Segura, Spanish National Research Council (CSIC), Murcia, Spain; ^10^Netherlands Institute for Vectors, Invasive Plants and Plant Health (NIVIP), Wageningen, Netherlands; ^11^Fera Science Ltd, York Biotech Campus, York, United Kingdom; ^12^European and Mediterranean Plant Protection Organization, Paris, France; ^13^Plant Sciences Unit, Institute for Agricultural, Fisheries and Food Research (ILVO), Merelbeke, Belgium; ^14^DLR Rheinpfalz, Institute of Plant Protection, Neustadt an der Weinstrasse, Germany; ^15^Bioversity International, Montpellier, France

**Keywords:** plant viruses and viroids, high throughput sequencing (HTS), biological characterization, plant health, regulatory agencies, Pest Risk Analysis (PRA), virus disease

## Abstract

The advances in high-throughput sequencing (HTS) technologies and bioinformatic tools have provided new opportunities for virus and viroid discovery and diagnostics. Hence, new sequences of viral origin are being discovered and published at a previously unseen rate. Therefore, a collective effort was undertaken to write and propose a framework for prioritizing the biological characterization steps needed after discovering a new plant virus to evaluate its impact at different levels. Even though the proposed approach was widely used, a revision of these guidelines was prepared to consider virus discovery and characterization trends and integrate novel approaches and tools recently published or under development. This updated framework is more adapted to the current rate of virus discovery and provides an improved prioritization for filling knowledge and data gaps. It consists of four distinct steps adapted to include a multi-stakeholder feedback loop. Key improvements include better prioritization and organization of the various steps, earlier data sharing among researchers and involved stakeholders, public database screening, and exploitation of genomic information to predict biological properties.

## Introduction

Advances in high-throughput sequencing (HTS) technologies and bioinformatic analyses have created new opportunities for the discovery and unbiased diagnosis of plant viruses and viroids (together referred to hereafter as viruses) (Massart et al., 2014). This exponential growth in the application of HTS technologies and the improvement of the bioinformatics algorithms have generated a steep increase in the discovery and publication of new sequences of viral origin (Shi et al., 2016; Chiapello et al., 2020; Edgar et al., 2022; Zayed et al., 2022; Rivarez et al., 2023).

A collective framework was published in 2017 to address the difficulties in assessing risks that these novel detections might pose. The framework aimed to suggest guidelines for researchers, policymakers, plant health authorities, and plant inspection services. It proposed an approach for prioritizing the biological characterization steps for newly identified plant viruses and evaluating their impact at biosecurity, commercial, regulatory and scientific levels (Massart et al., 2017). The first notification to the other plant health stakeholders in the framework was recommended after targeted methods (i.e., PCR or RT-PCR) confirmation of the novel virus detection by HTS. Then, if the novel virus was considered a phytosanitary priority, it was recommended to study its local prevalence and epidemiology (i.e., in the sampled field and surrounding area or in the batch of intercepted plants). Then a second communication with the regulatory authorities was proposed before further biological characterization of the novel virus, including fulfillment of Koch’s postulates, study of the mode of transmission, identification of potential vectors, evaluation of host range, symptomatology, and, if possible, global distribution. Finally, additional communication with authorities was recommended whenever considered relevant for the development of a Pest Risk Analysis (PRA) (Massart et al., 2017).

This framework was widely used to guide the characterization of newly identified plant viruses. However, recent reviews have shown that there is rarely a follow-up after the first report of novel viruses except for viruses that cause an immediate and obvious threat to production. Hou et al. (2020) reviewed 78 publications describing the discovery of novel viruses from 32 fruit tree species since 2011 and 933 citing publications. They observed interesting trends related to the characterization efforts carried out when publishing the discovery of a new fruit tree virus. The design of diagnostic primers and the completion of the genome sequence were done in more than 90% of the publications, underlining the importance but also the ease to obtain these two pieces of information. At large and local scales, infectivity assays and confirmation of a mixed infection were done in between 30 and 49% of the articles reviewed. Association with symptoms, studies on herbaceous indicators or other potential hosts, gene and genome diversity, latent infection and transmission assays were studied for 25% or less of the novel viruses.

Another publication by Rivarez et al. analyzed 53 published discovery and post-discovery studies on novel tomato viruses for the 2011–2020 period. It assessed how the framework by Massart et al. was fulfilled after the initial discovery (Rivarez et al., 2021). In most cases, a complete genome was provided and in approximately 80% of the articles, virus-specific primers were designed for diagnostic purposes. At the same time, more than 50% of the publications performed a local survey and gave information on the presence or absence of a co-infection with other viruses. However, less than 50% of the original publications studied the novel virus diversity, symptomatology or association with symptoms in field samples, infectivity on original and indicator hosts, or did a large-scale survey. A study on the natural host range of the novel virus was done only in less than 20% of the citing publications or post-discovery studies. Nevertheless, the framework’s criteria were fulfilled relatively quickly for novel viruses perceived as posing a considerable threat to crop production. For example, less than 4 years after the discovery of tomato brown rugose fruit virus (ToBRFV), which was discovered using non-HTS methods, 13 out of the 14 proposed characterization criteria had been fulfilled. In comparison, for tomato mottle mosaic virus and tomato necrotic stunt virus, that had been discovered through HTS, 11/14 criteria were fulfilled within 4–8 years.

**Figure 1:**
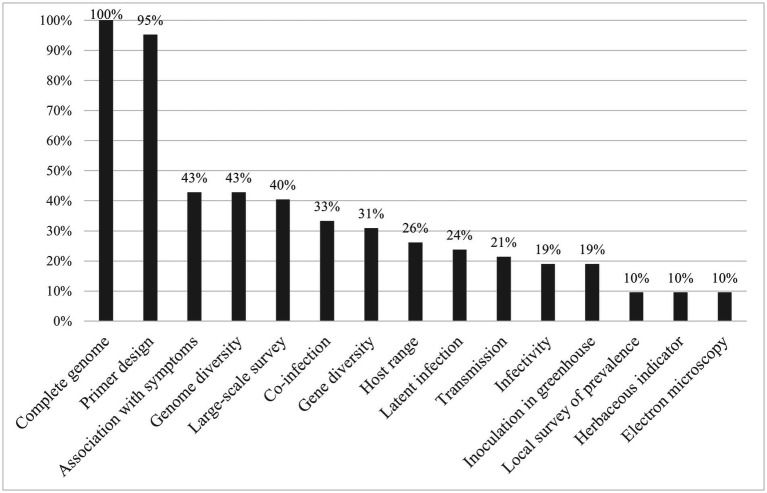
Percentage of newly identified *Poaceae* viruses for which data was developed for each characterization category, as defined by Hou et al. (2020).

Here, a similar analysis for 28 publications reporting 42 novel viruses identified by HTS in *Poaceae* was performed. The analysis is summarized in Figure 1 and further detailed in Supplementary material 1. Similar to the pattern observed for fruit tree and tomato viruses, the complete genome was published for all involved viruses, and in 95% of cases specific primers were designed. In contrast, further biological characterization studies such as the association with symptoms (43%) or electron microscopy (10%) were done much less often. Interestingly, gene and genome diversity were studied for 34 and 44% of the new *Poaceae* infecting viruses respectively, while for fruit tree viruses, they were mentioned in only 18 and 11% of cases and for tomato viruses in approximately 30 and 40%, respectively.

These three studies exemplify the exponential growth in plant viruses’ discovery due to HTS and the scarcity of biological characterization efforts for the identified novel viruses. The probable reasons for such an observation are the extended time and resources required for characterization experiments, including host range testing, large-scale surveys, and the technical difficulty of working with novel viruses for which little or no information is available. Nevertheless, there are some exceptions. For example, chestnut mosaic virus (ChMV) was identified by HTS technologies in symptomatic plants and was proposed as the potential causal agent of chestnut mosaic disease (ChMD). After obtaining a complete genome sequence from two chestnut disease sources, the genomes of ChMV were used to determine the phylogenetic relationships with other badnaviruses. New isolates were identified from publicly available chestnut HTS data. Incidence and genetic variability of ChMV were studied using samples from France and Italy (Marais et al., 2021). Another example is papaya virus X (PapVX), first identified in diseased papaya crops from northwest Argentina using HTS. Viral particles were confirmed with electron microscopy, and after obtaining a complete genome sequence, the genome organization and provisional taxonomic assignation were done. In addition, publicly available transcriptome datasets were also explored for other isolates of PapVX. The phylogenetic relationships were studied at nucleotide and amino acid levels for the RNA replicase (RdRp) and coat protein (CP) sequences and the complete genome. Mechanical inoculations were done to study the host range of PapVX, and a local survey in the northern region of Argentina was conducted to determine the distribution of the novel virus (Cabrera Mederos et al., 2022).

**Figure 2:**
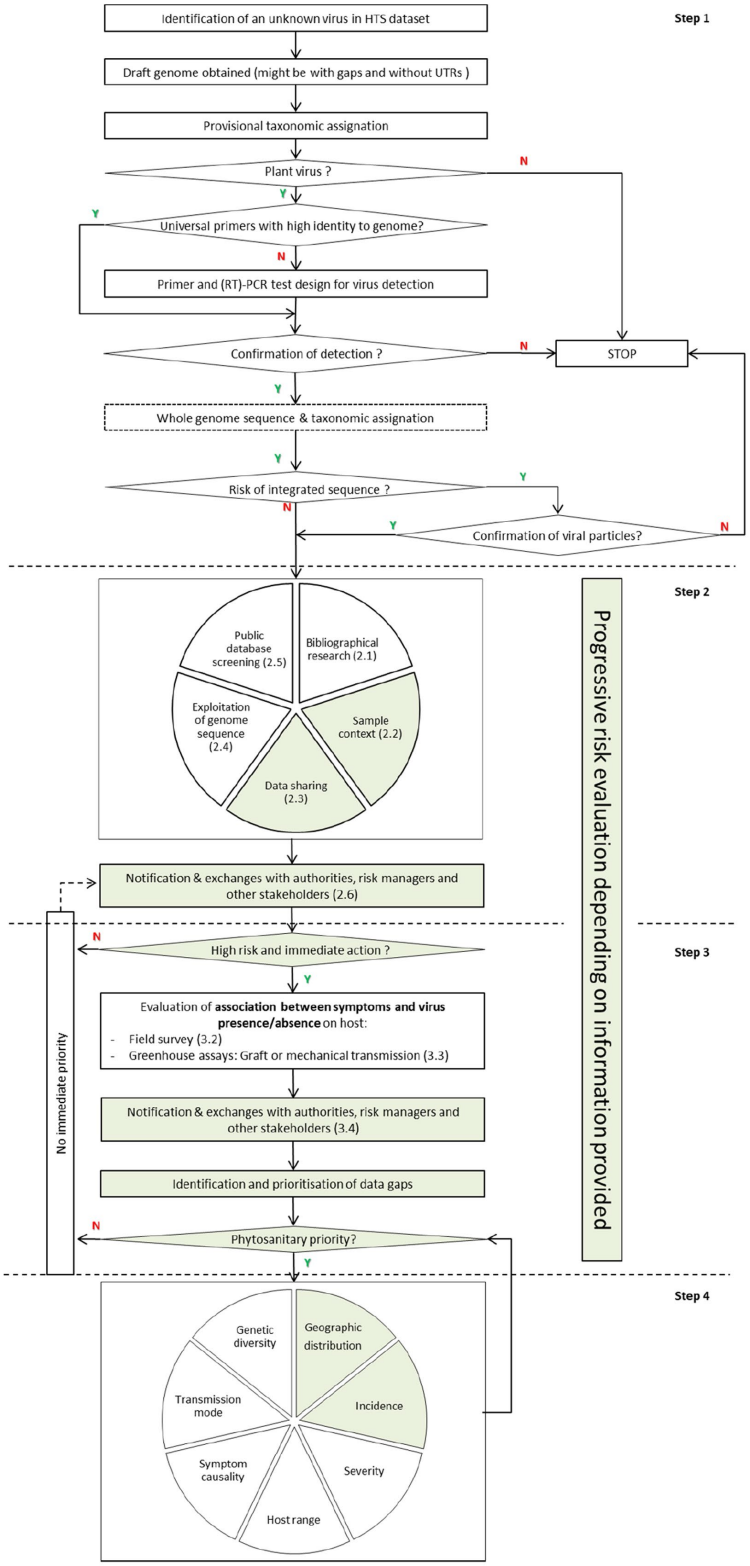
Proposed framework following the discovery of a novel virus or viroid. Y means positive response (yes) and N means negative response (no). Multi-stakeholders are involved in green-highlighted actions, and researchers in white-highlighted actions. Actions belonging to each step are separated with a dotted line, and numbers in brackets correspond to subchapters in the text.

Data on the geographic distribution, incidence, severity, symptomatology, host range, transmission mode, and genetic diversity of these novel viruses are necessary to support a proper risk assessment. Therefore, the previous framework is revised here to adapt it to the current rate of virus discovery through HTS, and add clarity on the prioritization of knowledge gaps (Figure 2). Furthermore, because of the recent reconsideration of the conceptual framework addressing the causal association between symptoms and the presence of a virus (Fox, 2020), this revision moves the evaluation of causal association at an earlier stage, as well as integrating the impact of HTS on plant health diagnostics and management (Adams et al., 2018; Olmos et al., 2018). The overall aim was to better adapt the framework to what is feasible, realistic, and efficient, while considering the limitation in time and resources that constrain the ability to fully characterize any newly discovered virus.

Data-driven virus discovery through scanning of large public sequencing datasets is a major recent development. Re-examining existing datasets for the presence of known and novel viruses has become accessible for virologists, through new web-based platforms like Serratus^1^ (Edgar et al., 2022), RVMT^2^ (Neri et al., 2022) and ViroidDB^3^ (Lee et al., 2022a,b). Nevertheless, virologists and plant health stakeholders should consider the consequences, not only benefits, of these data-driven virus discovery approaches (Lauber and Seitz, 2022).

In a short timeframe, these revolutionary high-throughput sequencing and data-driven approaches have extended the need to reconsider and adapt the current framework through a multi-stakeholder consultation. We thus propose an improved and adapted framework for plant health stakeholders, which could include researchers, policymakers, plant health authorities [also referred to as National Plant Protection Organizations (NPPOs)], plant inspection services, funding bodies, grower associations, technical extension services, seed traders, and breeding companies. It details the prioritization process to be followed for novel plant viruses and viroids identified by HTS technologies or datamining of HTS datasets.

## 1. Detection test, confirmation of detection and genome sequence

Importantly, international guidelines were proposed to improve the reliability of data generation by HTS technologies and their analysis by bioinformatics pipelines. These guidelines are generic and do not depend on the plant pest or pathogen being detected, sequencing protocol, or platform. These guidelines are advised to be implemented when applying HTS tests to detect viruses, whatever protocol is selected (EPPO PM 7/151 (1), 2022; Lebas et al., 2022; Massart et al., 2022).

In selected cases, the complete genome sequence is not obtained because of insufficient coverage (low read numbers), which can also depend on the library preparation protocol (ribosomal RNA depleted RNA, virion-associated nucleic acid (VANA), double-stranded RNA (dsRNA), small interfering RNA (siRNA), and total DNA with or without rolling circle amplification (RCA)) (Boonham et al., 2014; Hall et al., 2014; Roossinck et al., 2015; Claverie et al., 2019; Maclot et al., 2020) and the choice of the sequencing platform (i.e., Illumina sequencing, which generate short reads with large volume of sequences; or Oxford Nanopore Technologies, which generate longer but fewer reads) (Pfeiffer et al., 2018; Bester et al., 2021; Delahaye and Nicolas, 2021).

Nevertheless, once the draft genome of a potential novel virus was assembled, essential succeeding steps concern the annotation of the ORFs, the evaluation of percent pairwise identity, and phylogenetic relationships with known species. This information will be compared with the demarcation criteria established by the International Committee on Taxonomy of Viruses (ICTV) to evaluate if the assembled sequence belongs to a recognized species or to a new one. Even if the assembled genome lacks the UTRs or still has some gaps, the taxonomical position can still often be predicted (Lefkowitz et al., 2018). Furthermore, the construction of a phylogenetic tree should validate or help with the taxonomic assignation of the assembled genome into a genus or a family (Pagán, 2018). This work should be carried out with at least one representative from each closely-related genus and one outgroup. However, each case may be different, depending on the particular ICTV demarcation criteria applying to the virus under consideration. Thus, the analysis can/should be done using the RdRp, CP, or any other ORFs that are included in the relevant ICTV demarcation criteria. For example, for the family *Closteroviridae*, ICTV advises using RdRp, CP and HSP70h (70 kDA heat shock protein homolog) amino acid sequences to distinguish viral species (Candresse and Fuchs, 2020).

Provisional taxonomic assignment can be more complex if the newly discovered sequence is significantly divergent from known viruses or shows identity levels on the borderline with known taxa (Maclot et al., 2021). The viral sequences detected may correspond to a plant virus or any virus that infects an organism associated with the plant sample, such as bacteria, fungi, or insects (Al Rwahnih et al., 2011). For instance, the viral family *Partitiviridae* includes viruses that can infect plants, fungi, or protozoa (Vainio et al., 2018) so that determining whether a detected *Partitiviridae* infects the sampled plant or an associated organism may be complicated. Nevertheless, phylogenetic and taxonomic relationships to known viruses can facilitate the discrimination between plant, fungal, bacterial or insect viruses.

When detecting a potentially new viral species, the previous framework recommended confirmation of detection by a second test (Massart et al., 2017). This step remains essential, the common procedure being to use validated generic PCR tests if available. However, if a laboratory has a validated HTS test, this new finding could be tested using HTS (performing a new nucleic acid extraction) instead of PCR (Massart et al., 2022). For example, confirmation of detection of a novel virus is valid if the novel virus is identified in two independent laboratories using proper controls and validated HTS tests. Nevertheless, given the potential cross-contamination at each step of sample processing (i.e., sample collection, nucleic acid extraction, library preparation, and sequencing) (Rong et al., 2022), especially for viruses present in high concentration, it is advised to confirm the presence of the novel virus in the host using plant material of the original sample (back-up sample) for a new nucleic acid extraction.

Amplifying fragments of viruses/genomes using generic primers for a genus (or family) could also help in verifying the taxonomic assignment of the virus. It is possible that generic primers are not available or fail to amplify the novel virus, which can be because there are mismatches between the primer and the sequence to amplify, meaning that there will be a need to develop a more specific diagnostic tool that can detect the novel virus (Maree et al., 2018). The obtained amplicon may be further sequenced to confirm its viral origin. Usually, RT-PCR detection tests are developed using primers designed based on assembled sequences or a reference-based assembly to account for variability. If the genetic diversity of closely related viral species is well characterized, it is worth checking the scientific literature and aligning the existing sequences to evaluate the less variable ORF/regions within the taxonomic group as a way to design primers targeting a conserved genomic region to allow for detection of the most virus variants from the species. The ability of the designed primers to detect only the targeted new viral species and not related species should be checked by comparing their sequences to databases using primer BLAST, for example, or the genome alignments already developed. It is worth nothing that the specific test will be used as a diagnostic test in the following steps of the framework to complete the biological characterization of the new or poorly characterized virus (i.e., greenhouse assays and field surveys), as well as in managing the disease if the virus is causing symptoms to economically important crops. The test’s degree of specificity and sensitivity are therefore of prime importance.

When the full or near complete genome sequence is obtained from the initial HTS test, only the completeness of the genome sequence needs to be confirmed. For instance, viruses from the genus *Tenuivirus* have almost complementary 3′ and 5′ genome ends (Gaafar et al., 2021), which can provide an indication on genome completeness. Similarly, if the novel genome contains a 3′ poly-A tail or the assembled genome length is very similar to that of closely related viruses, it provides an indication that the UTRs are likely complete or only missing a few nucleotides (Kwibuka et al., 2021). However, if an incomplete genome assembly is obtained, it is a good practice to carry out additional analyses to complete the genome, such as iterative mapping of unassembled reads (Olmedo-Velarde et al., 2022). Nevertheless, obtaining the whole genome should not be the priority in an outbreak situation as long as primers can be designed for diagnostic purposes, and thus should not impede progression of the proposed framework. The genome sequence of the novel virus can be completed by filling the sequence gaps between contigs and determining the sequences of both extremities, usually using a rapid amplification of cDNA ends (RACE) (Marais et al., 2020; Maclot et al., 2021). Not all publications evaluate the genome completeness of a novel virus, and ICTV no longer recommends it as long as the complete set of ORFs are detected (Simmonds et al., 2017).

There are also specific cases that deserve particular attention, such as the case of some plant DNA viruses of families *Caulimoviridae* and *Geminiviridae*. These viruses can exist as endogenous viral elements integrated into the plant genome (i.e., endogenous pararetroviruses (EPRVs) for caulimoviruses or endogenous geminivirus-like (EGV) elements for geminiviruses) and/or as episomal forms that are contagious and can cause pathogenic infections (Sharma et al., 2020). Therefore, further investigation is necessary to verify whether the detected viral sequence corresponds to an infective episomal form or not. Endogenous viral sequences also represent a challenge for diagnostics and disease management, as a few endogenous viruses can revert to an infective episomal form (Rong et al., 2022). For example, several integrated banana streak viruses (BSV), tobacco vein clearing virus (TVCV), and petunia vein clearing virus (PVCV) can be activated to infectious episomal forms in specific plants hosts as a response to stress (Harper et al., 2002). Nonetheless, most endogenous viral sequences are not able to revert to episomal viruses, despite being transcribed. This could be verified by observing viral particles by electron microscopy (Chabannes and Iskra-Caruana, 2013) or with southern hybridization (Staginnus et al., 2007). Immunocapture PCR (IC-PCR) could be used if there are antibodies available, which, because the sequence in question belongs to a new virus, there probably are not (Le Provost et al., 2006). Rolling-Circle Amplification (RCA) is sometimes also used to distinguish between endogenous and episomal viral sequences (James et al., 2011). However, it is not recommended as it is not an absolute enrichment in circular sequences.

## 2. Contextual information gathering and notification to stakeholders

### 2.1. Bibliographical research on the biology of related viruses

After confirmation of the presence of a novel virus and its provisional taxonomic classification, the next step is the bibliographical research on the biology of related viruses (within the same family or genus). However, one should keep in mind that extrapolating the biological properties of a novel virus based on the viruses in the same taxa is associated with significant uncertainties.

At this stage, the main focus of the bibliographical research should be on (i) the putative modes of vertical and horizontal transmission and candidate vectors, if any, to assess the potential spread of the disease (Massart et al., 2017); (ii) the potential host range broadness and its botanical scope (Moury et al., 2017); (iii) the potential pathogenicity of the virus in its host(s), including symptomatology and the potential existence of helper or satellite viruses that may have an impact on symptoms and transmission, and (iv) in the case where broad-spectrum resistance is known against related viruses, the potential existence of resistance or tolerance to the novel virus in the identified host plant(s), keeping in mind that resistance or tolerance is often species-specific and, even with a broader spectrum, might still be lost for a closely related viral species. For example, the gene *Tm-2^2^* confers resistance against several tobamoviruses in tomatoes, but it does not protect against the newly discovered tomato brown rugose fruit virus (ToBRFV) (Hak and Spiegelman, 2021).

Even though information on closely related viruses can only give clues about the most probable mode of transmission of the novel virus or point to potential vectors, this information can be biased. For example, all members of the genus *Tenuivirus* are transmitted by a particular planthopper species, except maize yellow stripe virus (MYSV) that is transmitted by leafhoppers (Ammar et al., 2007; King et al., 2011). Viruses within the family *Geminiviridae* can be transmitted by whiteflies (*Begomovirus* genus), by leafhoppers (genera *Mastrevirus*, *Curtovirus*, *Becurtovirus*, *Mulcrilevirus*, and *Turncurtovirus*), by aphids (genus *Capulavirus*), or by treehoppers (genera *Topocuvirus* and *Grablovirus*) (Zerbini et al., 2017). Differences may also exist within a genus: torradoviruses are generally whitefly-transmitted (Vlugt et al., 2015), although some non-tomato infecting torradoviruses are aphid-transmitted (Rozado-Aguirre et al., 2016; Verbeek et al., 2017).

The information gathered from this bibliographical research will assist in elaborating possible epidemiological scenarios and hypotheses, from which further investigation on the host and vector range can be defined. This information can also help in formulating provisional tentative control measures included in the first notification to regulatory authorities, risk managers and other stakeholders. However, as mentioned before, this information is extrapolated from that of related viruses and should therefore be treated with caution.

### 2.2. Documentation of sample context

At this stage and to assist the risk assessment process, as much information as possible, whenever possible, should be collected regarding the original sample (or pool of samples) where the novel virus was detected. This includes the plant species and cultivar, sample accession number, description of the symptoms observed at the time of sampling, plant tissue collected, the viral status of the neighboring plants (if known either by onsite testing or from previous records), the incidence in the affected crop, other crops affected, recent meteorological conditions, sample collection date, geographical origin of the sample with specific map coordinates, and growth conditions of the plants (Massart et al., 2017). Collecting this information at the time of sampling can facilitate and minimize efforts later on. Additionally, the documentation could include the economic importance and geographical distribution of the crop species affected, globally or domestically (Kwibuka et al., 2021). Optionally, plants that are taxonomically related to the infected hosts, including other crops and wild plants that could potentially be threatened by the virus or be a reservoir or alternate host, could also be documented. This set of information could help make better preliminary assessment of the potential threat (García-Arenal and Zerbini, 2019; Hasiów-Jaroszewska et al., 2021; Rivarez et al., 2023). The information-gathering step, if done well, is critical and can decrease the burden during the submission of the dataset to public repositories such as the European nucleotide archive (ENA) or the sequence read archive (SRA) of GenBank.

### 2.3. Data sharing among research groups

Communication between stakeholders and the scientific community is essential for a quick decision-making process. Pre-publication data sharing between research groups that independently detected the novel virus is highly encouraged, because it can provide valuable information on the presence, distribution, host range, and impact of the novel virus (Koloniuk et al., 2018; Sõmera et al., 2019; Kwibuka et al., 2021; Temple et al., 2022). For example, actinidia virus X (AVX) was first reported as a novel virus infecting kiwifruit and blackcurrant, although it was later found to be synonymous with plantain virus X (PlVX) (Hammond et al., 2021); or potato virus V (PVV), which was confused with potato virus Y (PVY) since it caused similar symptoms when inoculated to PVY-sensitive cultivars (Fuentes et al., 2022). Nevertheless, data sharing is mainly done through informal contact between groups and is limited by the network of each researcher. The lack of communication and cooperation may lead to the multiplication of parallel efforts on the same issue (Giovani et al., 2020). Creating and improving networks, such as the global surveillance system (GSS), could enhance collaboration between stakeholders, nationally and internationally (Carvajal-Yepes et al., 2019). For example, a Euphresco (European phytosanitary research coordination) data-sharing project aims to improve pre-publication data-sharing approaches with a focus on documentation of sample context (Step 2.2) to explore similar findings from different research groups, thus providing access to distribution and host range data on novel virus detections. Data sharing could also be useful when research groups have many unpublished findings, which they may not be able to publish or disseminate on their own.

### 2.4. An unexplored path: exploitation of structural features from genomic sequence toward predictive sequence-to-function viral proteomics

In animal virology, many publications used machine learning approaches on databases of genomic features and biological properties from known viruses to predict the taxonomy or key biological properties of new viruses, such as host range and vector. Most of these approaches focus on nucleotide features like CG bias, CpG bias, di-codon, or dinucleotide bias (Young et al., 2020; Giovani et al., 2022). For example, dinucleotide bias was used to identify host reservoirs and vector candidates for mammalian RNA viruses (Babayan et al., 2018), to predict hosts of coronaviruses (Tang et al., 2015), or to identify the human or avian origin of influenza A viruses (IAV) using random forest analysis (Eng et al., 2016, 2017; Li et al., 2019).

Recent research used an original approach to identify new proteomic features potentially involved in plant virus-vector transmission, i.e., intrinsically disordered proteins/regions, and to understand how their biophysical properties and regulation might arise from these interactions (Tahzima et al., 2021). As a result, it was shown that most encoded plant virus proteins contain multiple disordered features that are phylogenomically preserved and can be associated with structural, bio-physical, and evolutionary strategies.

This opens a new focus for predicting the biological properties of the new plant virus from in-depth structural and functional analyses of protein sequences.

Nevertheless, interpreting all these features and results should still currently be done with much caution, given the uncertainty attached to such predictions and the sometimes limited accuracy of these databases. In the future, integrating these powerful emerging approaches to the framework could represent a significant step toward gathering relevant biological predictions from a genomic sequence. Therefore, it might ultimately support regulatory and phytosanitary decisions linked to discovering novel viruses.

### 2.5. Public database screening and consideration on careful use of related metadata

Valuable information can be gained by screening public databases of HTS data, such as the SRA of GenBank^4^, for the presence of newly identified or poorly characterized viruses (Hily et al., 2020). SRA is predicted to surpass 50 petabytes of data by 2023 [Sequence Read Archive (SRA) Data Working Group | DPCPSI, 2021; Katz et al., 2022] and mining such an enormous amount of information for virus presence previously required heavy computational power unaffordable for most virology laboratories as well as expertise in data science.

The recent development of a practical and user-friendly web-based interface called Serratus (Edgar et al., 2022) represents a major advancement toward a more generalized public database screening (see text footnote 1 for more details). Serratus uses a pre-screening strategy to look for viral RdRp motifs in SRA data (deposited until January 2020). It has the potential to provide hints about the host range or geographical distribution of specific RNA viruses present in the sequencing datasets. Following the pre-screening of SRA, Serratus provides a database of potential viral RdRp sequences (known and unknown), which is publicly available and can be used for exploratory and further diversity or phylogenetic analyses. This database also contains the link between each RdRp (SRA origin) and their associated “palmprint,”^5^ which is an RdRp “barcode” classified by taxonomy and clustered in operational taxonomic units (OTUs) with 90% identity threshold (Babaian and Edgar, 2021). Through Serratus, the SRA datasets deposited until January 2020 can be mined by looking for an RNA virus (via a family/Genbank/SRA_id search or a taxonomic tree exploration) or by searching for the sequence (protein or nucleic) of an RdRp using the palmID search tool.^6^

The palmID search tool allows finding the palmprint sequence within the provided RdRp and match it, with a minimal threshold of pairwise identity, to palmprint sequences in the palmprint database. The palmprint OTU is necessary to avoid heavy computational requirements, although it lowers the confidence in the results, thus only giving hints to the presence of the target virus, which needs further validation. Thus, the sequencing reads of the identified SRA dataset should be reanalyzed using existing bioinformatic approaches to confirm the presence of the virus of interest. Supplementary material 2 presents a practical example of the additional information that can be gained by using palmID for an emerging virus (physostegia chlorotic mottle alphanucleorhabdovirus).

Nevertheless, Serratus has some limitations, mainly when the virus of interest is not detected from public SRA datasets. Even in case of detection, the verification step (assembly and mapping) is time-consuming and a computational burden, and sometimes inefficient, depending for example on virus representation and on sample identity (i.e., fragmented genome or pooled sample). Another limitation is the potential misassignment of the host, in particular when the new finding involves a metagenomic dataset with unexpected virome content (i.e., a plant virus found in a human clinical dataset or animal viruses found in plant datasets). In addition, metadata information such as the host or the country of origin of the sequenced material should also be investigated, keeping in mind that the metadata may not be accurate. If biological material is still available, contacting the authors may allow the confirmation of the detection. It should be stressed that there could also be some implications for trade and related policies when relying on SRA mining for reporting the detection of pathogens in a country where it is not currently known to be present. In such a case, the conduct of confirmatory tests in the wet lab should be encouraged and given a very high priority. The ethics of reporting the presence of a pathogen in a territory without prior notification to its NPPO should also be considered. Whenever possible, it is advised to contact the dataset’s original authors and notify the country’s NPPO before the publication of a new country record.

### 2.6. Notification and exchanges with other stakeholders

Based on the studies described in steps 2.1 to 2.5, researchers should have a better idea of whether a newly identified virus might threaten plant health and whether the new finding(s) should be reported to other stakeholders. However, it is crucial not to overburden relevant stakeholders with non-relevant information that might raise unnecessary concerns. For example, the detection of a plant virus, belonging to a family of pathogenic viruses with high horizontal transmission rates, on a sample of a critical crop should be communicated as soon as possible to the NPPO. In contrast, detecting a partitiviridae in an asymptomatic wild plant has lower significance and, therefore, priority. The participation of plant virology experts is therefore crucial at this stage to support well-informed decision making. As mentioned before, when a virus is considered a potential threat to plant health, researchers should report the finding to the relevant NPPO, engage in discussions with risk managers and assist them in efforts to determine if the novel virus should be considered a priority, and whether immediate action (i.e., destruction of consignment) or specific management measures (i.e., disinfection or rouging) should be taken and to evaluate whether further research is needed. Given the potential impact of the management decision taken by the NPPO, the uncertainties associated with the discovery of the novel virus and its potential impacts should be highlighted in a transparent fashion.

Further on, if the NPPO analysis confirms the potential threat following consultations, the main challenge for scientists is to efficiently characterize the biological properties through short, mid- and long-term strategies. This is while creating appropriate communication channels with the regulatory authorities and other stakeholders including grower associations, technical extension centers, or seed companies (Massart et al., 2017; Fiallo-Olivé and Navas-Castillo, 2019).

## 3. Evaluation of the association between symptoms and virus presence

### 3.1. General background

After the first notification to the regulatory agencies, if a novel virus is considered a priority or has potential risks, further evaluation of the association between symptoms and virus presence must be carried out via field surveys or greenhouse assays. Field surveys and greenhouse assays can provide helpful information regarding symptomatology, infectivity, causal association, virus genetic diversity, geographic distribution, incidence, host range, transmission mode, and disease severity, as discussed in step 4. Nevertheless, at this stage, it is essential to focus the survey’s aim and assays on the symptom(s) causation issue if the novel virus is considered a priority. These efforts should not be hindered by those toward the completion of previous steps, since obtaining a complete genome sequence might be time-consuming and could delay the needed surveys or assay actions.

In conventional plant pathology approaches, when trying to establish a causal association between a disease and a pathogen, causation is demonstrated by isolating the putative pathogenic agent and subjecting it to the experimental demonstration of Koch’s postulates (Rivers, 1937; Evans, 1976). Nevertheless, this strategy has downsides because not all diseases are caused by a single pathogen, and pathogen complexes, timing of infection and influence of abiotic factors may also play a role in disease development. There are examples of situations in which causation could not be shown by fulfilling Koch’s postulates, such as different virus strains causing a variable array of symptoms on the same host (Blystad et al., 2015), environmental conditions affecting the disease (Fraile and García-Arenal, 2016), the importance of the time passed after the infection (Chikh-Ali et al., 2020), or pathogens in an active mixed infection (Murphy and Bowen, 2006).

In recent years, there has been an ongoing discussion among researchers to find possible alternative and systematic approaches that overcome the limitations of Koch’s postulates in plant virology (Di Serio et al., 2018; Fox, 2020). These efforts follow numerous previous attempts (Ehrlich, 1913; Rivers, 1937; Huebner, 1957; Hill, 1965; Johnson and Gibbs, 1974; Falkow, 1988; Evans, 1991; Fredericks and Relman, 1996). As a consequence of the most recent efforts, a simplification of criteria needed to establish causal association was proposed, mainly focusing on four key considerations: experimental evidence, the strength of the relationship, consistency of the relationship, and a binary evaluation of coherence and plausibility (Fox, 2020). A simplified hierarchical approach was thus proposed when considering a causal relationship in plant virology based on four criteria: (i) experimental, which complies with Koch’s third postulate; (ii) strength, which is based on field/glasshouse observations, confirmation of a single pathogen infection by HTS and statistical analysis, considering the prevalence of the virus and eventually co-infecting species on both symptomatic and asymptomatic individuals; (iii) consistency, following the same principle and approach as for the “strength” criteria but adding the variable of multiple geographic locations and over time; and (iv) coherence and plausibility, which account for any confounding factors and similar effects that have been reported in other pathosystems.

### 3.2. Field surveys

Surveys at small- and/or large scales allow a better understanding of the key factors associated with a disease. These epidemiological field surveys should include both symptomatic and asymptomatic plants since an asymptomatic individual could be in the incubation or latent phase at the time of sampling. In field surveys, caution should be taken in generating and analyzing data on the virus variability to ensure that the viral populations in crops and weeds are sufficiently similar to support the hypothesis of the role of weeds as a potential reservoir. Neighboring plants from the same or different species that present similar symptoms may also be tested for presence or absence of the novel virus, without the need for viral enrichment extraction protocols, and instead using commercial extraction kits or even crude extracts (Massart et al., 2009). Because mixed infections are common, field surveys can be based on targeted tests, such as RT-PCR, or HTS. Whenever possible, especially for viruses detected in perennial hosts, the survey should be carried out at different time points of the year on the same individual plants and on more than one plant tissue. This is because there are seasonal fluctuations of the viral titer that can impact the detection of the virus and because viruses in many woody hosts show an uneven distribution in host tissues (Katsiani et al., 2018; Tahzima et al., 2019; Beaver-Kanuya and Harper, 2021). This multiple sampling approach allows more time for the disease and symptoms to develop on previously healthy-looking but already infected plants. In addition, following the virus spread over the years in the fields where it has been detected can provide information on how rapidly the prevalence is changing. Depending on the interim risk assessment conclusions, plants positive for the novel virus may represent a risk requiring their prompt removal to avoid further spread of the disease. If supported by statistical analyses, surveys allow sound evaluation of the association between virus presence and disease development (Adams et al., 2014).

### 3.3. Greenhouse assays

Greenhouse assays are commonly used to assess symptom causality and symptomatology. Since mixed infection can occur in the source material, working on single viral species during greenhouse assays is essential. In many cases, particularly with graft inoculation, these techniques cannot separate viruses in a mixed infection. One solution to this limitation is the use of infectious clones during the biological characterization of the virus as proposed in the previous framework (Massart et al., 2017). Nevertheless, constructing infectious clone can be a complex and time-consuming step that is not possible for all plant viruses. Other approaches include use of differential hosts (i.e., with known virus resistance or preference to viruses), vector or seed transmission, and thermotherapy. The novel virus can be inoculated to indicator plants, host plant candidates, or other cultivars of the original host species by mechanical or graft inoculation (Wu et al., 2020). Identifying the mode(s) of transmission of the virus would facilitate the greenhouse assays, which the bibliographical research (elaborated in step 2.1) may give clues before being experimentally tested. Other factors to consider when designing a greenhouse assay are availability of detection tests for the novel virus, host range, choice of indicator plant, developmental stage of the plant during the inoculation, greenhouse climate conditions, availability of space, and greenhouse biosecurity or biosafety level required for the experiment, as well as ensuring that the host and indicator plants are virus- or pathogen-free (da Silva et al., 2020; Panno et al., 2020; EPPO PM 7/153 (1), 2022).

### 3.4. Notification and exchanges with other stakeholders

The additional biological information obtained will progressively feed the risk evaluation. It should be shared with stakeholders via, for example, a reporting system or *ad hoc* meetings with relevant plant health authorities. A meeting between the involved parties can be organized to analyze the new information obtained since the last notification (step 2.3), to assess the status of the novel virus as a pest, and if it needs to be regulated (EPPO, 2012; IPPC ISPM 11, 2019; IPPC ISPM 2, 2019; IPPC ISPM 21, 2021). This discussion will help identify further data gaps and to prioritize the research focus as the assessment moves forward. At this point, exchanges with the authorities, risk managers, and stakeholders will allow re-evaluation of risks posed by the virus and reach a provisional decision on its phytosanitary status.

## 4. Completion of data gaps to strengthen the risk evaluation process

**Figure 3:**
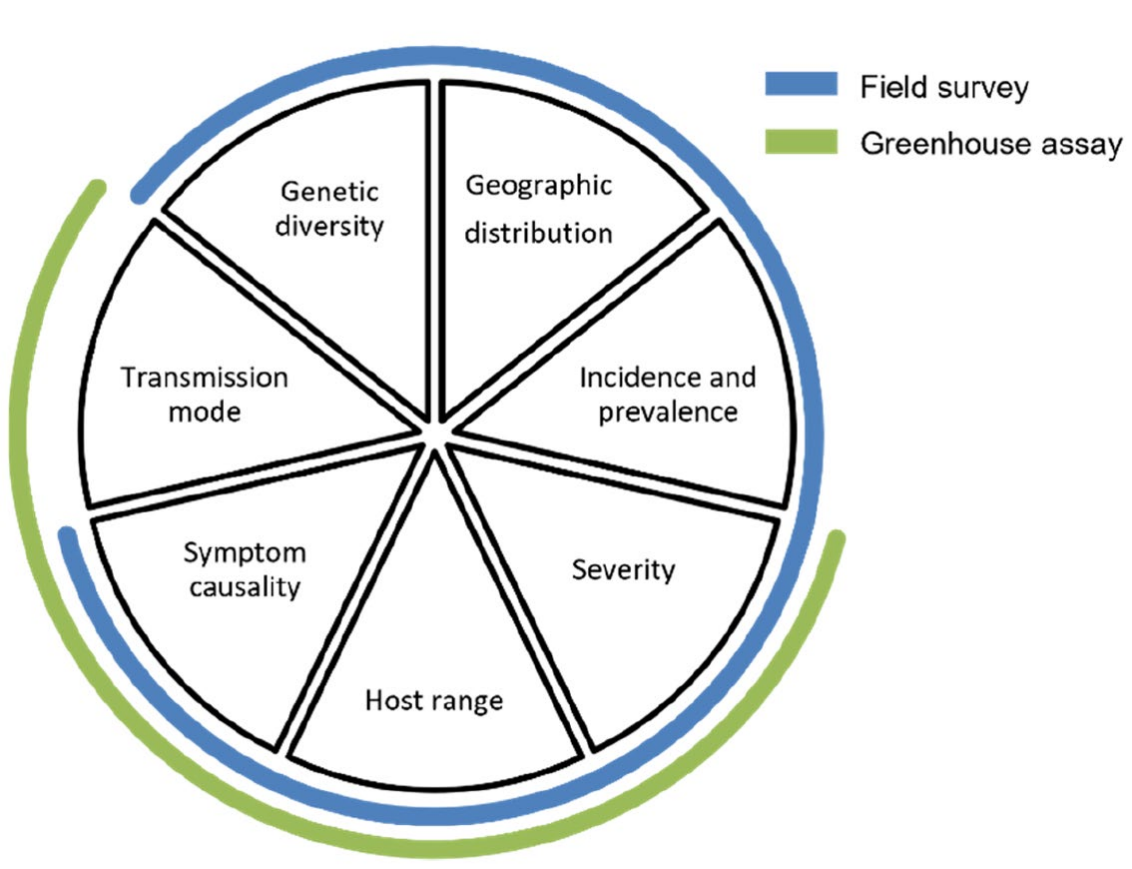
Pie chart diagram summarizing the data gaps to be filled in step 4 of the framework (adapted from Figure 2).

At this point, knowledge/data gaps can remain uncompleted resulting in significant uncertainties, with an ensuing need for strengthening and refining the risk evaluation for the virus, especially if it is still considered a phytosanitary priority. During discussions with stakeholders and plant health authorities, these data gaps, which can be of various kinds, should be identified and filled through further field surveys (small- or large-scale) and greenhouse assays. As shown in Figure 3, a well-designed field survey can provide missing information on genetic diversity, geographic distribution, incidence and prevalence, severity of the disease (if any), host range, and symptom causality (if any); and a well-designed greenhouse assay can provide additional information on severity of the disease, host range, symptom causality, and transmission mode. Additionally, the researcher may focus on filling more specific data gaps such as the effect of mixed infections, susceptibility of different cultivars and other economically important host plants, the effect of other biotic/abiotic stressors and, if possible, variability of pathogenicity between isolates (Chinnaraja and Viswanathan, 2015).

It is possible that despite the efforts described in step 3, causation issues were not solved. In that case, further field surveys or greenhouse assays informed by the partial or negative outcome of the early efforts can be envisioned to fill the remaining data gaps on disease causation. This is necessary to assess the priority status of novel viruses and demonstrate their role in disease development. Once the association between presence/absence of the virus and symptoms in the host is confirmed, the aim is to determine the severity of the disease symptoms on host plant species and to estimate the potential yield and economic losses due to said disease, which can be done by (i) surveys to assess the impact on infected plants (Gent et al., 2004), and (ii) greenhouse (or field) inoculation assays (Nancarrow et al., 2021). For practical experimental reasons, the impact on yield and quality might prove challenging to estimate in greenhouse trials. Noticeably, not all viruses will cause a disease, while some may even be beneficial for the host (Roossinck, 2015; Aguilar et al., 2017). Severity and symptoms may vary depending on the other viruses infecting the host or environmental and other external factors (Bertazzon et al., 2017).

It is also essential to study the potential spread of the virus and its geographic distribution, to assess the situation’s urgency and the measures to be taken, particularly when considering the need to restrict plant commodities’ circulation. For this, large-scale field surveys (both nationally and internationally) are necessary, as well as collecting symptomatic and asymptomatic plants and test them with the diagnostic protocols designed in step 1 or with generic tests such as HTS, to better determine the presence of mixed infections. Ideally, this large-scale evaluation could be supported by a network of collaboration with other stakeholders (i.e., plant virologists or plant inspection services) that could facilitate the exchange of samples.

Whenever possible, sampling of geographically and phylogenetically related wild and domesticated plant species should be considered to expand the knowledge of the potential host range, study its prevalence and identify potential reservoirs, since earlier efforts may have provided incomplete information (Wintermantel et al., 2009). However, it is worth noting that the host range is never fully known as novel natural hosts are frequently described after the initial discovery. Closely related crop species of known hosts can potentially become hosts themselves (Xing et al., 2020), thus maybe experimental evolution assays or untargeted virome surveys could be conducted.

Accounting for the genetic variability of a viral population when designing the experiments is essential to improve the inclusiveness of detection tests and study the origin, dynamics, evolution, and phylogenetic relationships of the novel virus (Kutnjak et al., 2014; Kawakubo et al., 2021). This diversity can be studied through whole genome sequencing of isolates obtained from field surveys or by partial genome sequencing of a specific genomic region showing a level of variability.

Knowing the primary transmission mechanism of a virus is advised to properly design a successful greenhouse assay, as well as to evaluate risks and design an efficient disease/pest management strategy. Although difficult to accomplish, from a risk assessment perspective it is important to know about all transmission mechanisms as it can influence the fitness of the novel virus, selection pressures driving resistance and tolerance genes in the host, and viral population structure (Stewart et al., 2005; Pagán et al., 2014; da Silva et al., 2020).

## 5. Conclusion

The recent reviews of Hou et al. (2020) and Rivarez et al. (2021), as well as the similar analysis done here for *Poaceae*, highlighted the need for a revision of the previous characterization framework. Conducting similar systematic reviews in other crops might give additional insights on what is the current landscape on plant virus characterization after a first identification in an HTS dataset. Essentially, this revision emphasizes adapting a progressive feedback approach during the risk evaluation process, highlights the growing importance of database mining, proposes as a keystone the disease causal association, and underlines the importance and benefits of data and effort sharing, as well as the advantages to collaborate with other researchers. It is worth noting that this is not a substitution for any decision-support scheme for a pest risk analysis or pest categorization but a complementary document, especially useful for cases where the virus is considered as non-priority or where communication with plant health authorities may be more limited. Researchers may be overwhelmed with the number of findings in HTS datasets, or have a lack of resources and time, so this revision serves as an outline of the prioritization steps to go further than the genomic and molecular characterization of a novel virus, which will produce more useful and practical information for plant health authorities and producers/grower associations. In the long term, advances in artificial intelligence, machine learning technologies and bioinformatic tools will facilitate the process of characterization of a novel virus and reduce the resources and time needed. For example, SRA mining tools have the potential to complement conventional global surveys although a discussion around the technical and ethical considerations of using such methods should be held between the scientific community and stakeholders. Similar to the aim of the previous framework, this work should be regularly adapted by authorities to help rationalize and accelerate decisions on the most relevant actions at the different stages of virus discovery and characterization. In addition, this characterization framework can also be adapted for different countries not only for plant viruses but also for animal viruses and even other pathogens.

## Data availability statement

The original contributions presented in the study are included in the article/Supplementary material, further inquiries can be directed to the corresponding author.

## Author contributions

NF, MK, AyM, MRi, JR, FS, CoT, MA, NB, TC, AF, YH, DK, ArM, FP, MRa, and SM wrote the first draft. NF did the later work to expand and improve the text. MB, IS, RT, ChT, TW, and SM revised the text. All authors contributed to the article and approved the submitted version.

## Funding

This work was supported by the European Union’s Horizon 2020 Research and Innovation Program under grant agreement no. 773139 (VALITEST) and under the Marie Skłodowska-Curie grant agreement no. 813542 (INEXTVIR), by the CGIAR Genbank platform and by the Germplasm Health Unit (GHU) improvement initiative.

## Conflict of interest

AF was employed by Fera Science Ltd. AyM and YH were employed by Abiopep S.L.

The remaining authors declare that the research was conducted in the absence of any commercial or financial relationships that could be construed as a potential conflict of interest.

## Publisher’s note

All claims expressed in this article are solely those of the authors and do not necessarily represent those of their affiliated organizations, or those of the publisher, the editors and the reviewers. Any product that may be evaluated in this article, or claim that may be made by its manufacturer, is not guaranteed or endorsed by the publisher.

## Footnotes

^1^www.serratus.io

^2^www.riboviria.org

^3^www.viroids.org

^4^https://www.ncbi.nlm.nih.gov/sra

^5^https://github.com/rcedgar/palmdb

^6^www.serratus.io/palmid
