# Supplementary material for: Managing the deluge of newly discovered plant viruses and viroids: an optimized scientific and regulatory framework for their characterization and risk analysis
Source: Front Microbiol. 2023 May 30;14:1181562. doi: 10.3389/fmicb.2023.1181562 (PMC10265641; doi:10.3389/fmicb.2023.1181562)
Supplement: Supplementary file 1 [file Data_Sheet_1.pdf]

SUPPLEMENTARY MATERIAL Nº1

The present review provides in depth analysis of information provided by a representative set of publications reporting the discovery of new viruses infecting *Poaceae* species. The 28 publications reporting newly discovered *Poaceae* viruses were screened according to several categories of information adapted from Hou et al. (2020): genome completeness, primer design, association with symptoms, gene and genome diversity, large-scale survey, co-infection, host range, latent infection, transmission, infectivity, inoculation in greenhouse, local survey of prevalence, and herbaceous indicator (Table 1).

A total of 42 complete genomes of new viral species detected by HTS were reported, among of which, 40 (95%) have been confirmed by designing of specific primers, amplifying the targeted genomic region and sequencing of the amplicon. For panicum ecklonii associated virus (PeaV), lolium erenne associated virus (LpaV), holcus lanatus associated virus (HlaV), and stipagrostic associated virus (SaV) only the complete genome and specific primers were reported (Richet et al., 2019). Furthermore, the diversity of the genome or specific genes was analysed for 43% (18) and 31% (13) of new viruses respectively, including, for example, rice latent virus 1 (RLV1) and 2 (RLV2) (Kraberger et al., 2017) and barley yellow dwarf virus OYV (BYDV-OYV) (Sõmera et al., 2021). This diversity analysis is sometimes impossible to achieve. Indeed, one publication, having discovered wheat umbra-like virus (WULV) and wheat-associated vipovirus (WaVPV), did not report information on genome or gene diversity because only a single isolate was detected (Redila et al., 2021).

This analysis showed that majority of samplings was carried out on symptomatic wild and cultivated *Poaceae* species (17/28 studies). While for 7 studies, samples were collected regardless of whether they were symptomatic or asymptomatic. Co-infection has been reported for 33% of viruses and 13% were present as a single infection, including for example maize yellow dwarf virus (MYDV)-like polerovirus (Massawe et al., 2018), maize-associated pteridovirus (MaPV) (Read et al., 2019a) and morogoro maize-associated virus (MMaV) (Read et al., 2019b). For the rest of the viruses, it was not stated in the publications. On the other hand, latent infections were reported for 24% of new viral species, for example for sorghum mastrevirus-associated alphasatellite (SMasA) (Claverie et al., 2020). Nevertheless, results for silvergrass cryptic virus 1 (SgCV-1) are not yet conclusive because no obvious symptoms were observed and its impact in mixed infections is unclear (Costa et al., 2022). Large-scale field surveys were conducted for 40% (17) of the viruses, including, for example, bromus-associated circular DNA viruses 1-4 (BasCV1-4) and trifolium-associated circular DNA virus 1 (TasCV-1) (Kraberger et al., 2015).

Inoculation to herbaceous indicators was carried out for 4 (10%) viruses, although it was only successfull for tall oatgrass mosaic virus (TOgMV) (Hassan et al., 2014). For wheat yellow stunt associated betaflexivirus (WYSaBV) herbaceous indicators remained symptomless and it was not detected by RT-PCR at 30 days post inoculation (Fu et al., 2021). Association with symptoms was demonstrated for 43% (18) of viruses: wheat yellow stunt-associated betaflexivirus (WYSaBV), sugarcane striate virus (SCStV) (Boukari et al., 2017), wheat dwarf India virus, maize streak Réunion virus (Pande et al., 2012), TOgMV, stentaphrum nepovirus (SteNV) (Tran et al., 2021), festuca stripe-associated virus (FSaV) (Gaafar et al., 2021), wheat virus Q (WVQ), maize-associated totivirus (MATV) (Chen et al., 2016), wheat stripe mosaic virus (WhSMV), sugarcane umbra-like virus (SULV) (Tahir et al., 2021), alopecurus myosuroides partitivirus 1 and 2 (AMPV1-2), alopecurus myosuroides varisacovirus (AMVV1), wheat leaf yellowing associated virus (WLYaV) (Zhang et al., 2017), wheat yellow

striate virus (WYSV) (Liu et al., 2018), wheat yellow dwarf virus (WYDV) (Guo et al., 2022) and miscanthus yellow fleck virus (Bolus et al., 2020).

Studies to identify alternative hosts have been carried out for 11 virus species (26%). A single additional host has been identified for 3 viruses: *Digitaria didactyla* for SteNV (Tran et al., 2021), *Avena sativa* for TOgMV (Hassan et al., 2014) and *Urochloa maxima* for sorghum arundinaceum associated virus (SAAV) (Claverie et al., 2019). For the other viruses, several host plants were identified. Transmission pathways were reported for 9 viral species (21%): potential natural vectors have been identified for wheat dwarf india virus (*Psammotettix sp.*) and wheat stripe mosaic virus (*Polymyxa graminis*) (Kumar et al., 2012; Valente et al., 2019). poaceae Liege nepovirus A was successfully transmitted through seed (Maclot et al., 2021). In addition, soil transmission of wheat virus Q was successful although the exact pathway was not identified (Kondo et al., 2021). Alopecurus myosuroides partitivirus 1 and 2 were mentioned to be transmitted by pollen (Sabbadin et al., 2017). Only 3 and 10 publications completed the discovery with local or large-scale survey, respectively.

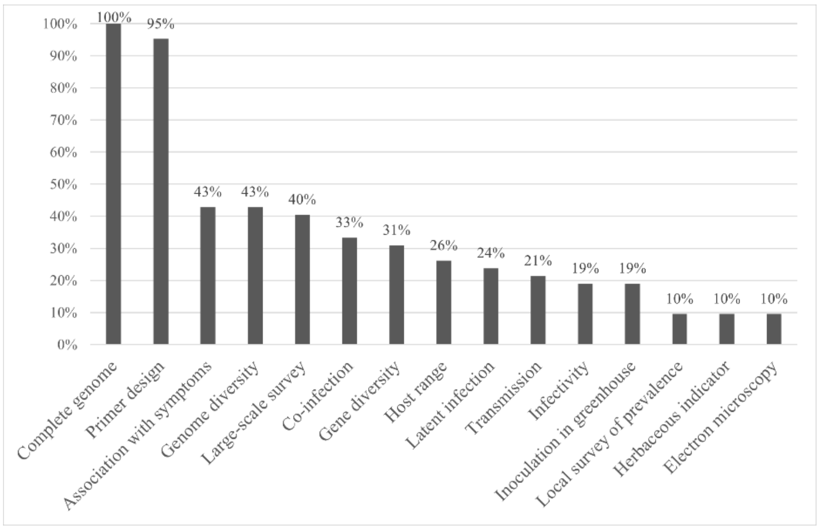

Figure 1. Percentage of newly identified *Poaceae* viruses for which data was developed for each characterization category, as defined by Hou et al. (2020).

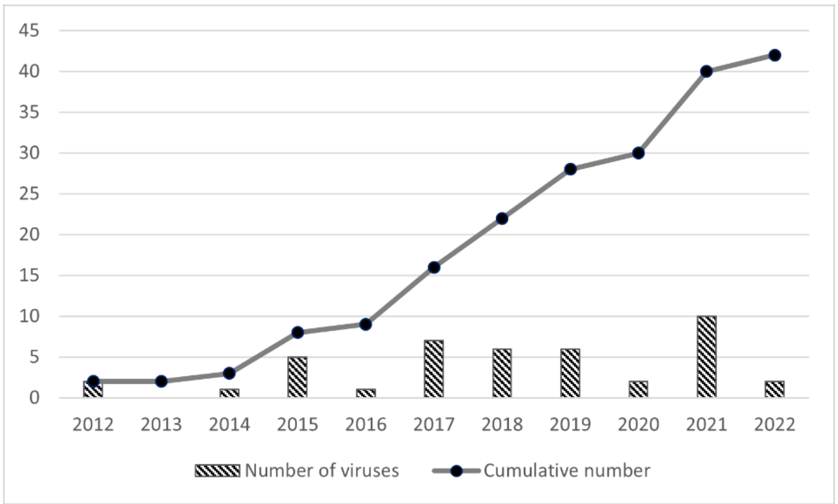

Figure 2. Evolution of the number of novel viruses infecting *Poaceae* discovered by HTS since 2012.

Table 1. Studies done for each newly identified *Poaceae* viruses for each characterization category, as defined by Hou et al. (2020). Boxes in blue represent actions taken and boxes in white actions not taken.

	Publication	Year	Virus	Complete genome	Primer design	Association with symptom	Genome diversity	Large-scale survey	Co-infection	Gene diversity	Host range	Latent infection	Transmission	Infectivity	Inoculation in greenhouse	Local survey of prevalence	Herbaceous indicator	Electron microscopy
1	Tran et al	2021	Stentaphrum nepovirus (SteNV)	■	■	■	■	■	■		■					■		■
2	Gaafar et al	2021	Festuca stripe-associated virus (FSaV)	■	■	■								■	■			■
3	Richet et al	2018	Stipagrostis associated virus (SaV)	■	■													
4	Richet et al	2018	Panicum ecklonii associated virus (PeaV)	■	■													
5	Richet et al	2018	Lolium perenne associated virus (LpaV)	■	■													
6	Richet et al	2018	Holcus lanatus associated virus (HlaV)	■	■													
7	Maclot et al	2021	Poaceae Liege virus 1 (PoLV1)	■	■						■	■	■			■		
8	Maclot et al	2021	Poaceae Liege nepovirus A (PolNVA)	■	■						■	■	■			■		
9	Fu et al	2021	Wheat yellow stunt-associated betaflexivirus (WYSaBV)	■		■	■		■	■				■	■		■	■
10	Kondo et al	2021	Wheat virus Q (WVQ)	■	■	■	■		■	■	■	■	■	■	■			
11	Kraberger et al	2017	Rice latent virus 1 (RLV1)	■	■		■	■				■						
12	Kraberger et al	2017	Rice latent virus 2 (RLV2)	■	■		■	■				■						
13	Sabbadin et al	2017	Alopecurus myosuroides partitivirus 1 (AMPV1)	■	■	■		■	■		■	■	■					
14	Sabbadin et al	2017	Alopecurus myosuroides partitivirus 2 (AMPV2)	■	■	■		■	■		■	■	■					
15	Sabbadin et al	2017	Alopecurus myosuroides varisacovirus (AMVV1)	■	■	■		■	■		■	■	■					
16	Boukari et al	2017	Sugarcane striate virus (SCStV)	■	■	■	■	■										
17	Chen et al	2016	Maize-associated totivirus (MATV)	■	■	■												
18	Claverie et al	2019	Eleusina indica associated virus (EIAV)	■	■		■			■	■							
19	Claverie et al	2019	Sorghum arundinaceum associated virus (SAAV)	■	■		■			■	■							
20	Claverie et al	2019	Melinis repens associated virus (MeRAV)	■	■		■			■	■							
21	Claverie et al	2020	Sorghum mastrevirus-associated alphasatellite (SMasA)	■	■		■		■			■						
22	Somera et al	2021	Barley yellow dwarf virus OYV (BYDV-OYV)	■	■		■		■	■								
23	Kumar et al	2012	Wheat dwarf India virus (WDIV)	■	■	■							■	■	■			
24	Massawe et al	2018	Mayze yellow dwarf virus (MYDV)-like polerovirus	■	■			■	■									
25	Pande et al	2012	Maize streak Réunion virus (MSRV)	■		■	■			■								
26	Read et al	2019	Maize-associated pteridovirus (MaPV)	■	■				■									
27	Read et al	2019	Morogoro maize-associated virus (MMaV)	■	■			■	■									
28	Valente et al	2019	Wheat stripe mosaic virus (WhSMV)	■	■	■	■	■		■			■					
29	Hassan et al	2014	Tall oatgrass mosaic virus (TOgMV)	■	■	■		■			■			■	■		■	■
30	Kraberger et al	2015	Bromus-associated circular DNA virus 1 (BasCV1)	■	■		■	■		■								
31	Kraberger et al	2015	Bromus-associated circular DNA virus 2 (BasCV2)	■	■		■	■		■								
32	Kraberger et al	2015	Bromus-associated circular DNA virus 3 (BasCV3)	■	■		■	■		■								
33	Kraberger et al	2015	Bromus-associated circular DNA virus 4 (BasCV4)	■	■		■	■		■								
34	Kraberger et al	2015	Trifolium-associated circular DNA virus 1 (TasCV-1)	■	■		■	■		■								
35	Tahir et al	2021	Sugarcane umbra-like virus (SULV)	■	■	■			■									
36	Costa et al	2022	Silvergrass cryptic virus 1 (SgCV-1)	■	■							■				■		
37	Redila et al	2021	Wheat umbra-like virus (WULV)	■	■				■									
38	Redila et al	2021	Wheat-associated vipovirus(WaVPV)	■	■													
39	Zhang et al	2017	Wheat leaf yellowing associated virus (WLYaV)	■	■	■								■	■		■	
40	Liu et al	2018	Wheat yellow striate virus (WYSV)	■	■	■							■	■	■			■
41	Guo et al	2022	Wheat yellow dwarf virus (WYDV)	■	■	■		■	■									
42	Bolus et al	2020	Miscanthus yellow fleck virus (MYFV)	■	■	■								■	■		■	■
			Percentage	100%	95%	43%	43%	40%	33%	31%	26%	24%	21%	19%	19%	10%	10%	10%

Bolus, S., Malapi-Wight, M., Grinstead, S.C., Fuentes-Bueno, I., Hendrickson, L., Hammond, R.W., Mollov, D., 2020. Identification and characterization of Miscanthus yellow fleck virus, a new polerovirus infecting Miscanthus sinensis. PLOS ONE 15, e0239199. https://doi.org/10.1371/journal.pone.0239199

Boukari, W., Alcalá-Briseño, R.I., Kraberger, S., Fernandez, E., Filloux, D., Daugrois, J.-H., Comstock, J.C., Lett, J.-M., Martin, D.P., Varsani, A., Roumagnac, P., Polston, J.E., Rott, P.C., 2017. Occurrence of a novel mastrevirus in sugarcane germplasm collections in Florida, Guadeloupe and Réunion. Virol. J. 14, 146. https://doi.org/10.1186/s12985-017-0810-9

Chen, S., Cao, L., Huang, Q., Qian, Y., Zhou, X., 2016. The complete genome sequence of a novel maize-associated totivirus. Arch. Virol. 161, 487–490. https://doi.org/10.1007/s00705-015-2657-y

Claverie, S., Ouattara, A., Hoareau, M., Filloux, D., Varsani, A., Roumagnac, P., Martin, D.P., Lett, J.-M., Lefeuvre, P., 2019. Exploring the diversity of Poaceae-infecting mastreviruses on Reunion Island using a viral metagenomics-based approach. Sci. Rep. 9, 12716. https://doi.org/10.1038/s41598-019-49134-9

Claverie, S., Varsani, A., Hoareau, M., Filloux, D., Roumagnac, P., Martin, D.P., Lefeuvre, P., Lett, J.-M., 2020. Sorghum mastrevirus-associated alphasatellites: new geminialphasatellites associated with an African streak mastrevirus infecting wild Poaceae plants on Reunion Island. Arch. Virol. 165, 1925–1928. https://doi.org/10.1007/s00705-020-04685-5

Costa, L.C., Hu, X., Malapi-Wight, M., O'Connell, M., Hendrickson, L.M., Turner, R.S., McFarland, C., Foster, J., Hurtado-Gonzales, O.P., 2022. Genomic characterization of silvergrass cryptic virus 1, a novel partitivirus infecting Miscanthus sinensis. Arch. Virol. 167, 261–265. https://doi.org/10.1007/s00705-021-05294-6

Fu, S., Zhang, T., He, M., Sun, B., Zhou, X., Wu, J., 2021. Molecular characterization of a novel wheat-infecting virus of the family Betaflexiviridae. Arch. Virol. 166, 2875–2879. https://doi.org/10.1007/s00705-021-05175-y

Gaafar, Y.Z.A., Rabenstein, F., Zia, A., Gaafar, A.-R.Z.A., Ziebell, H., 2021. Molecular characterisation of a new tenuivirus from Festuca sp. Virus Res. 304, 198509. https://doi.org/10.1016/j.virusres.2021.198509

Guo, M., Yuan, X., Wu, N., Liu, Y., Wang, X., 2022. Complete genome sequence of a novel wheat-infecting polerovirus associated with yellowing dwarf disease in China. Arch. Virol. 167, 983–987. https://doi.org/10.1007/s00705-022-05360-7

Hassan, M., Širlová, L., Vacke, J., 2014. Tall oatgrass mosaic virus (TOgMV): a novel member of the genus Tritimovirus infecting Arrhenatherum elatius. Arch. Virol. 159, 1585–1592. https://doi.org/10.1007/s00705-013-1905-2

Hou, W., Li, S., Massart, S., 2020. Is There a "Biological Desert" With the Discovery of New Plant Viruses? A Retrospective Analysis for New Fruit Tree Viruses. Front. Microbiol. 11, 2953. https://doi.org/10.3389/fmicb.2020.592816

Kondo, H., Yoshida, N., Fujita, M., Maruyama, K., Hyodo, K., Hisano, H., Tamada, T., Andika, I.B., Suzuki, N., 2021. Identification of a Novel Quinvirus in the Family Betaflexiviridae That Infects Winter Wheat. Front. Microbiol. 12, 2219. https://doi.org/10.3389/fmicb.2021.715545

Kraberger, S., Farkas, K., Bernardo, P., Booker, C., Argüello-Astorga, G.R., Mesléard, F., Martin, D.P., Roumagnac, P., Varsani, A., 2015. Identification of novel Bromus- and Trifolium-associated circular DNA viruses. Arch. Virol. 160, 1303–1311. https://doi.org/10.1007/s00705-015-2358-6

Kraberger, S., Geering, A.D.W., Walters, M., Martin, D.P., Varsani, A., 2017. Novel mastreviruses identified in Australian wild rice. Virus Res. 238, 193–197. https://doi.org/10.1016/j.virusres.2017.07.003
Kumar, Jitendra, Singh, S.P., Kumar, Jitesh, Tuli, R., 2012. A novel mastrevirus infecting wheat in India. Arch. Virol. 157, 2031–2034. https://doi.org/10.1007/s00705-012-1359-y
Liu, Y., Du, Z., Wang, H., Zhang, S., Cao, M., Wang, X., 2018. Identification and Characterization of Wheat Yellow Striate Virus, a Novel Leafhopper-Transmitted Nucleorhabdovirus Infecting Wheat. Front. Microbiol. 9.
Maclot, F.J., Debue, V., Blouin, A.G., Fontdevila-Pareta, N., Tamisier, L., Filloux, D., Massart, S., 2021. Identification, molecular and biological characterization of two novel secovirids in wild grass species in Belgium. Virus Res. 198397. https://doi.org/10.1016/j.virusres.2021.198397
Massawe, D.P., Stewart, L.R., Kamatenesi, J., Asiimwe, T., Redinbaugh, M.G., 2018. Complete sequence and diversity of a maize-associated Polerovirus in East Africa. Virus Genes 54, 432–437. https://doi.org/10.1007/s11262-018-1560-5
Pande, D., Kraberger, S., Lefeuvre, P., Lett, J.-M., Shepherd, D.N., Varsani, A., Martin, D.P., 2012. A novel maize-infecting mastrevirus from La Réunion Island. Arch. Virol. 157, 1617–1621. https://doi.org/10.1007/s00705-012-1314-y
Read, D.A., Featherston, J., Rees, D.J.G., Thompson, G.D., Roberts, R., Flett, B.C., Mashingaidze, K., Pietersen, G., Kiula, B., Kullaya, A., Mbega, E., 2019a. Characterization and detection of maize-associated pteridovirus (MaPV), infecting maize (Zea mays) in the Arusha region of Tanzania. Eur. J. Plant Pathol. 154, 1165–1170. https://doi.org/10.1007/s10658-019-01703-4
Read, D.A., Featherston, J., Rees, D.J.G., Thompson, G.D., Roberts, R., Flett, B.C., Mashingaidze, K., Pietersen, G., Kiula, B., Kullaya, A., Mbega, E.R., 2019b. Molecular characterization of Morogoro maize-associated virus, a nucleorhabdovirus detected in maize (Zea mays) in Tanzania. Arch. Virol. 164, 1711–1715. https://doi.org/10.1007/s00705-019-04212-1
Redila, C.D., Prakash, V., Nouri, S., 2021. Metagenomics Analysis of the Wheat Virome Identifies Novel Plant and Fungal-Associated Viral Sequences. Viruses 13, 2457. https://doi.org/10.3390/v13122457
Richet, C., Kraberger, S., Filloux, D., Bernardo, P., Harkins, G.W., Martin, D.P., Roumagnac, P., Varsani, A., 2019. Novel circular DNA viruses associated with Apiaceae and Poaceae from South Africa and New Zealand. Arch. Virol. 164, 237–242. https://doi.org/10.1007/s00705-018-4031-3
Sabbadin, F., Glover, R., Stafford, R., Rozado-Aguirre, Z., Boonham, N., Adams, I., Mumford, R., Edwards, R., 2017. Transcriptome sequencing identifies novel persistent viruses in herbicide resistant wild-grasses. Sci. Rep. 7, 41987. https://doi.org/10.1038/srep41987
Sõmera, M., Massart, S., Tamisier, L., Sooväli, P., Sathees, K., Kvarnheden, A., 2021. A Survey Using High-Throughput Sequencing Suggests That the Diversity of Cereal and Barley Yellow Dwarf Viruses Is Underestimated. Front. Microbiol. 12, 673218. https://doi.org/10.3389/fmicb.2021.673218
Tahir, M.N., Bolus, S., Grinstead, S.C., McFarlane, S.A., Mollov, D., 2021. A new virus of the family Tombusviridae infecting sugarcane. Arch. Virol. 166, 961–965. https://doi.org/10.1007/s00705-020-04908-9
Tran, N.T., Teo, A.C., Crew, K.S., Campbell, P.R., Thomas, J.E., Geering, A.D.W., 2021. Genome sequence and geographic distribution of a new nepovirus infecting

Stenotaphrum secundatum in Australia. Virus Res. 305, 198554. https://doi.org/10.1016/j.virusres.2021.198554

Valente, J.B., Pereira, F.S., Stempkowski, L.A., Farias, M., Kuhnem, P., Lau, D., Fajardo, T.V.M., Nhani Junior, A., Casa, R.T., Bogo, A., da Silva, F.N., 2019. A novel putative member of the family *Benyviridae* is associated with soilborne wheat mosaic disease in Brazil. Plant Pathol. 68, 588–600. https://doi.org/10.1111/ppa.12970

Zhang, P., Liu, Y., Liu, W., Cao, M., Massart, S., Wang, X., 2017. Identification, Characterization and Full-Length Sequence Analysis of a Novel Polerovirus Associated with Wheat Leaf Yellowing Disease. Front. Microbiol. 8.
