# Supplementary material for: Managing the deluge of newly discovered plant viruses and viroids: an optimized scientific and regulatory framework for their characterization and risk analysis
Source: Front Microbiol. 2023 May 30;14:1181562. doi: 10.3389/fmicb.2023.1181562 (PMC10265641; doi:10.3389/fmicb.2023.1181562)
Supplement: Supplementary file 2 [file Data_Sheet_2.pdf]

SUPPLEMENTARY MATERIAL Nº2

Physostegia chlorotic mottle virus (PhCMoV) is an alphanucleorhabdovirus discovered by high throughput sequencing on Lamiaceae (Menzel *et al.*, 2018). Subsequently, the virus was detected in nine European countries on nine different host plants belonging to seven plant families. Its presence was associated with severe symptoms on cultivated tomato plants (Temple *et al.*, 2022).

In the frame of its characterization, Serratus (Edgar *et al.*, 2022) was used to scan for the presence of PhCMoV sequences in public RNASeq SRAs. Briefly, a palmID derived from the virus L protein (RNA dependent RNA polymerase, RdRp) was used to search the Serratus palmID database. More than 2660 hits were obtained ranging from 93% palm-identity (E-value=3.5e-79) to 37% palm-identity (4.7e-13). To confirm the presence of PhCMoV, SRA datasets with the best palmID hits (above 90% identity) were downloaded and their reads mapped on the PhCMoV reference genome (NC_055466) using Bowtie2 (Langmead and Salzberg, 2012; Langmead *et al.*, 2009) on Galaxy (Galaxy Version 2.4.5) with default parameters. Such a confirmation of Serratus results should always be performed and the filters (mapping or serratus PalmID) should be adapted according to the diversity in the family of the virus under consideration.

The presence of PhCMoV was detected in five SRA datasets belonging to two bioprojects (PRJNA636634 and PRJNA449559). Associated metadata provided information as to the identity of the sequenced samples, and where and when these samples had been collected and sequenced. Interestingly, these "detections" added considerable knowledge on PhCMoV by expanding its distribution range to a new continent (China, Asia). A high number of reads (> 94939 reads) of four biosamples mapped on the PhCMoV genome with a high genome coverage (>99.4%) (see Table 1). In addition, the virus was found associated with two new plant species (*Polemonium pulcherrimum* and *Lavandula angustifolia*). The detection of PhCMoV in *P. pulcherrimum* (biosample: SAMN15153850: 305133 PhCMoV reads, Table 1) was important because it could represent an expansion of the virus host range to a new family *(Polemoniaceae*), while *L. angustifolia* does not represent an expansion of the host range because it belongs to the family *Lamiaceae* (already known). The *P. pulcherrimum* sample was collected in a botanical garden in Germany. Furthermore, to better understand the evolution and distribution of the virus, the recovered PhCMoV genomes could be included in an updated PhCMoV phylogenetic tree.

Nevertheless, such findings should be taken with caution, especially in instances where the discovery of the virus of interest in an SRA dataset is unexpected. It is thus recommended to contact the researchers responsible for the bioproject and confirm the information regarding the origin of the plant material and sample context, including potential presence of symptoms. In addition, testing by RT-PCR the original biological material, if any is remaining, would allow to confirm the presence of the virus in a new host or new geographic location.

Without the Serratus interface, it would be very cumbersome to scan the huge number of SRA datasets publicly available. Such an approach allows the serendipitous identification of novel host-virus combinations as it was unlikely that *P. pulcherrimum* would be considered as a potential plant host for PhCMoV during the bibliographical research (Step 2.1). But as always when important diagnostic results are at stake, care should be taken when trying to interpret such results.

Table 1. Number of reads that map to the reference of PhCMoV (NC_055466) for each SRA dataset and their associated metadata.

Bioproject (NCBI)	Run_id (NCBI)	Biosample_id (NCBI)	Palm_id	Plant Family	Host plant	Country of origin	Number of mapped reads	% identity NC_055466	horizontal Coverage
PRJNA636634	SRR12002078	SAMN15153850	u3491	Polemoniaceae	Polemonium pulcherrimum	Germany	305193	97%	100%
PRJNA449559	SRR6980628	SAMN08716985	u3491	Lamiaceae	Lavandula angustifolia	China	94939	99%	99.6%
PRJNA449559	SRR6980626	SAMN08717030	u3491	Lamiaceae	Lavandula angustifolia	China	103126	99%	99.5%
PRJNA449559	SRR6980629	SAMN08716984	u3491	Lamiaceae	Lavandula angustifolia	China	154723	99%	99.6%
PRJNA449559	SRR6980627	SAMN08717026	u3491	Lamiaceae	Lavandula angustifolia	China	103673	99%	99.4%

References

Edgar, R.C., Taylor, J., Lin, V., Altman, T., Barbera, P., Meleshko, D., Lohr, D., Novakovsky, G., Buchfink, B., Al-Shayeb, B., Banfield, J.F., de la Peña, M., Korobeynikov, A., Chikhi, R., Babaian, A. (2022). Petabase-scale sequence alignment catalyses viral discovery. *Nature*, **602**: 142–147.

Langmead, B., Salzberg, S.L. (2012). Fast gapped-read alignment with Bowtie 2. *Nature Methods*, **9**: 357–359.

Langmead, B., Trapnell, C., Pop, M., Salzberg, S.L. (2009). Ultrafast and memory-efficient alignment of short DNA sequences to the human genome. *Genome Biology*, **10**: R25.

Menzel, W., Richert-Pöggeler, K., Winter, S., Knierim, D. (2018). Characterization of a nucleorhabdovirus from Physostegia. *Acta Horticulturae*, **1193**: 29–38.

Temple, C., Blouin, A.G., De Jonghe, K., Foucart, Y., Botermans, M., Westenberg, M., Schoen, R., Gentit, P., Visage, M., Verdin, E., Wipf-Scheibel, C., Ziebell, H., Gaafar, Y.Z.A., Kutnjak, D., Vučurović, A., Rivarez, M.P.S., Richert-Pöggeler, K.R., Ulrich, R., Zia, A., Yan, X.-H., Massart, S. (2022). Biological and genetic characterization of Physostegia chlorotic mottle virus in Europe based on host range, location, and time. *Plant Disease*.
